# Epigenetic modifications at DMRs of placental genes are subjected to variations in normal gestation, pathological conditions and folate supplementation

**DOI:** 10.1038/srep40774

**Published:** 2017-01-18

**Authors:** Beenish Rahat, Aatish Mahajan, Rashmi Bagga, Abid Hamid, Jyotdeep Kaur

**Affiliations:** 1 Department of Biochemistry, Postgraduate Institute of Medical Education and Research, Chandigarh 160012, India; 2 Department of Obstetrics and Gynecology, Postgraduate Institute of Medical Education and Research, Chandigarh 160012, India; 3 Cancer Pharmacology Division, Indian Institute of Integrative Medicine, 180001, Jammu, India

## Abstract

Invasive placentation and cancer development shares many similar molecular and epigenetic pathways. Paternally expressed, growth promoting genes (*SNRPN, PEG10* and *MEST*) which are known to play crucial role in tumorogenesis, are not well studied during placentation. This study reports for the first time of the impact of gestational-age, pathological conditions and folic acid supplementation on dynamic nature of DNA and histone methylation present at their differentially methylated regions (DMRs). Here, we reported the association between low DNA methylation/H3K27me3 and higher expression of *SNRPN*, *PEG10* and *MEST* in highly proliferating normal early gestational placenta. Molar and preeclamptic placental villi, exhibited aberrant changes in methylation levels at DMRs of these genes, leading to higher and lower expression of these genes, respectively, in reference to their respective control groups. Moreover, folate supplementation could induce gene specific changes in mRNA expression in placental cell lines. Further, *MEST* and *SNRPN* DMRs were observed to show the potential to act as novel fetal DNA markers in maternal plasma. Thus, variation in methylation levels at these DMRs regulate normal placentation and placental disorders. Additionally, the methylation at these DMRs might also be susceptible to folic acid supplementation and has the potential to be utilized in clinical diagnosis.

The proper development of fetus throughout pregnancy is regulated by efficient placental growth. Similar to cancer metastasis, placental development involves proliferation and invasion of placental trophoblasts into normal maternal uterus and hence display a phenotype resembling cancerous cells. There are many shared molecular mechanisms between invasive placentation and metastasis, especially in terms of the factors which enhance growth^1^,^2^,^3^. These similarities also appear at key epigenetic mechanisms^4^. Additionally the placental growth seems to be enhanced by paternally expressed genes, which are known to show growth promoting phenotype^5^. Studies have suggested major influence of paternal genome in placental development^6^,^7^ and higher occurrence of paternally expressed genes in placenta^8^. These genes are generally regulated by cis-acting differentially methylated regions (DMRs)^9^. These regions are usually also associated with differential histone marks^10^. Such differential epigenetic marks at DMRs of these genes lead to their differential expression via a complex sequence of events, however, if the DMR is also the promoter of that specific gene it directly silences the methylated allele.

Disruption of these epigenetic marks at DMRs result in abnormal gene expression leading to major phenotypic changes^11^, associated with developmental deformities^12^, placental disorders^13^,^14^ and malignancies^15^. Considering the similarity between cancer and placentation, it is likely that placental epigenome too is liable to alterations during advancing gestation, pathological conditions and external factors like availability of nutrients etc.

Disease pathologies associated with inappropriate gene expression are quite often related to aberrantly functioning placenta. Preeclampsia, which is associated with placental dysfunction, is one such pathology which might be associated with defective gene expression^16^, leading to poor invasion of endovascular trophoblasts^17^,^18^ and abnormal remodeling of maternal spiral arteries^19^. Paternally expressed genes have also been recently reported to play an important role in the pathogenesis of preeclampsia^20^. These genes might be of special importance in the development of preeclampsia as these genes are known to control trophoblastic invasion and placental growth^21^.

Hydatidiform mole (a type of gestational trophoblastic diseases, GTDs) is another placental disorder, believed to be associated with a higher ratio of paternal to maternal genome arising due to fertilization of either normal or enucleated egg with two spermatozoa (dispermy) or a diploid spermatozoon^22^. Thus, the elevated ratio of paternal genome which triggers the aberrant expression of paternally expressed genes has been associated with the pathogenesis of molar pregnancy^23^. Although, hydatidiform mole is usually benign, it may progress to a highly invasive and malignant form of trophoblastic tumor known as choriocarcinoma, characterized by hemorrhagic metastasis attributed to altered vascular permeability^24^.

Small nuclear ribonucleoprotein-associated protein N (*SNRPN*), paternally expressed gene 10 (*PEG10*) and mesoderm-specific transcript (*MEST*) are three paternally expressed genes with known growth promoting phenotype in general. Studies have also predicted their possible role in normal placental development^25^,^26^,^27^. *SNRPN* is a small nuclear ribonucleoprotein complex involved in pre-mRNA processing events like tissue specific alternative splicing^28^. The expression of *PEG10* is predominently high in mouse and human placenta and its deletion has been associated with early embryonic lethality^26^, thus emphasizing the importance of *PEG10* in development of placenta^29^. *MEST* is a member of the [α/β] hydrolase fold family and play a prominent role in angiogenesis placental trophoblast and decidua. It is highly expressed in the placenta and its loss leads to placental growth restriction^30^. It is candidate gene involved in retardation of primordial growth observed in Silver–Russell syndrome (SRS)^31^. The DMRs of *SNRPN*^32^, *PEG10*^33^ and *MEST*^34^ are localized in their promoter regions.

Folic acid being a key source of the one carbon group required to methylate DNA^35^, is generally recommended preconceptionally during early pregnancy^36^. DNA methylation is an epigenetic modification critical to normal development of placenta and regulation of gene expression. Therefore, folate supplementation can directly affect gene expression, which emphasizes the importance of the analysis of the effect of folic acid supplementation on expression of these genes.

Previous studies from our lab^37^ and others have highlighted the importance of epigenetic modifications in regulating the differential expression of various genes in placenta. Based on this background information we aimed to analyze the effect of advancing gestation, placental dysfunction and nutritional supplementation on epigenetic mechanisms at the DMRs within the promoter region of *SNRPN*, *PEG10* and *MEST* and their effect on their relative expression. We also investigated the potential of the DMRs of these genes as fetal DNA epigenetic markers in maternal plasma, for their future use in prenatal diagnosis and the diagnosis of pregnancy related disorders.

## Results

### mRNA expression of imprinting genes in normal and pathological pregnancies

**Figure 1:**
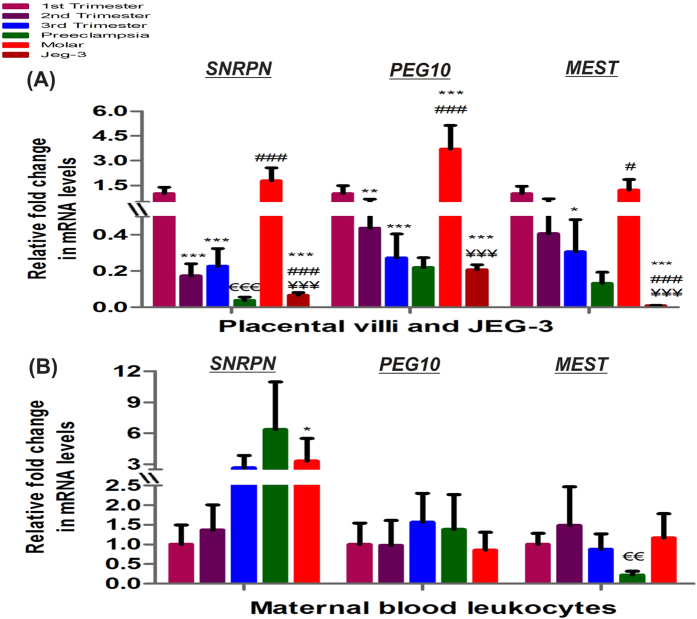
Relative fold change in mRNA of imprinting genes normalized with GAPDH. Relative mRNA expression among (**A**) placental villous samples and JEG-3 cells (**B**) maternal blood leukocytes [*p < 0.05, **p < 0.01, ***p < 0.001 *vs* 1^st^ trimester, ^#^p < 0.05, ^###^p < 0.001 *vs* 2^nd^ trimester, ^€€^p < 0.01, ^€€€^p < 0.001 *vs* 3^rd^ trimester and ^¥¥¥^p < 0.001 *vs* molar]. The data is presented as mean of the observed fold change ± SEM, n = 30 per group.

Genes known to show imprinting phenomenon play important role during placental development and its related pathologies. We analyzed the mRNA expression of three imprinting genes *SNRPN*, *PEG10* and *MEST* in physiological first, second and third trimester groups and placental disorders (preeclampsia and molar pregnancy) and JEG-3 cells (choriocarcinoma cell line) (Fig. 1A and B). The mRNA expression of all the three imprinting genes were observed to reduce with gestation, decreasing by 4.4–5.7 fold after midgestation (p < 0.001) in case of *SNRPN*, while decreasing by 2.3- (p < 0.01) and 3.6- (p < 0.001) folds in second trimester and third trimester respectively for *PEG10* and by 3.3 fold (p < 0.05) in third trimester for *MEST* with respect to first trimester placental villi (Fig. 1A). mRNA estimation of *SNRPN*, *PEG10* and *MEST* in maternal blood leukocytes revealed non-significant change within normal gestational groups (Fig. 1B). The analysis of mRNA expression of these genes in the preeclampsia and GTD groups suggested the possible association between abnormal expression of these genes and the development of these disorders. Preeclamptic villi exhibited lower expression of all three imprinting genes in reference to normal third trimester placental villi, however it was significantly reduced by 5.9 fold (p < 0.001) only in case of *SNRPN*. Similarly, *MEST* mRNA levels were also decreased by 3.9 fold (p < 0.01) in maternal blood leukocytes of preeclamptic women relative to its control group. Development of molar pregnancy and choriocarcinoma were observed to be related with reverse trend in the mRNA expression of these imprinting genes. Molar villi was observed to show the higher expression of these genes with respect to second trimester villi being raised by 10.2 fold (p < 0.001), 8.4 fold (p < 0.001) and 3 fold (p < 0.05) for *SNRPN, PEG10* and *MEST*, respectively. The expression of *SNRPN* was also raised by 3.3 fold (p < 0.05) in molar maternal blood leukocytes with respect to first trimester group. In JEG-3 cells the mRNA expression of these imprinting genes was observed to be significantly lower being reduced by 15.1- and 26.8- fold (p < 0.001), 4.8- and 17.7- fold (p < 0.001) and 107.6- and 131.6- fold (p < 0.001) respectively for *SNRPN*, *PEG10* and *MEST* as compared to normal first trimester and molar villi.

Pearson correlation analysis depicted significantly high positive correlation between mRNA expression levels of these imprinting genes among all five placental villi groups and JEG-3 cells, Pearson correlation coefficient r being 0.94 (p < 0.01), 0.96 (p < 0.01) and 0.87 (p < 0.05) for *SNRPN* vs *PEG10*, *SNRPN* vs *MEST* and *PEG10* vs *MEST* respectively.

### DMRs of imprinting genes show variation in methylation with gestation and pathological conditions

To study the role of DNA methylation in mediating the differential mRNA expression of *SNRPN*, *PEG10* and *MEST* during physiological advancing gestation and pregnancy related complications, we estimated CpG site DNA methylation at DMRs/promoters of these genes in placental villi samples, maternal blood cells and placental cell lines.

**Figure 2:**
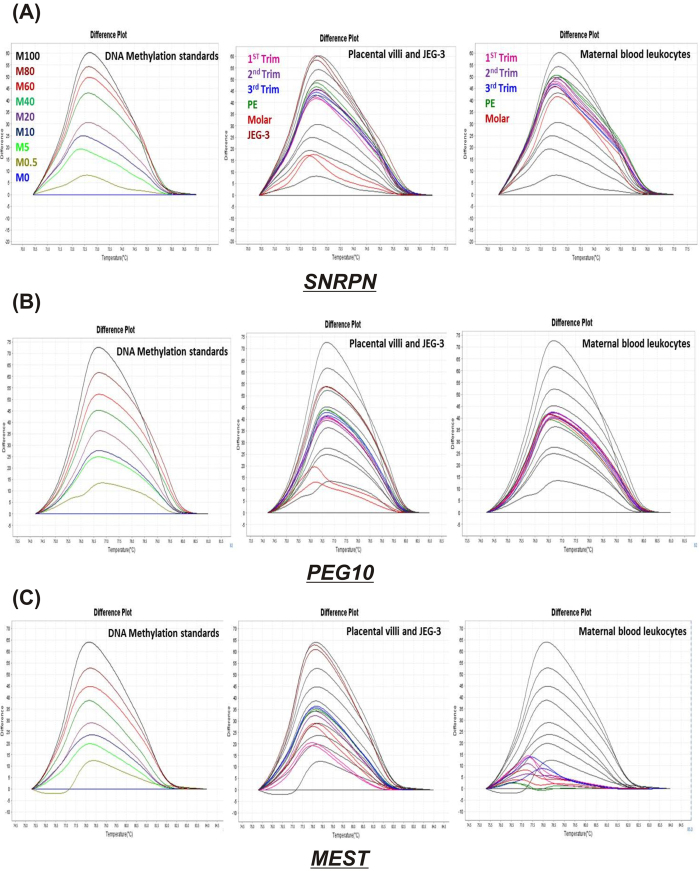
DNA methylation at DMRs of imprinting genes as predicted by HRM software. HRM difference plots for (**A**) *SNRPN* (**B**) *PEG1*0 (**C**) *MEST*. Each representing three difference plots: first one for methylation standards in different colors (M100% - M0%, which stands for DNA methylation standards with 100 to 0% methylation) normalized to the 0%-methylated standard DNA, second and third difference plots for few selected samples from placental villous groups & JEG-3 cells and maternal leukocyte groups represented in different colors and methylated standard curve of M100 to 0% represented as black curves. Note: “Trim” stands for trimester.

**Figure 3:**
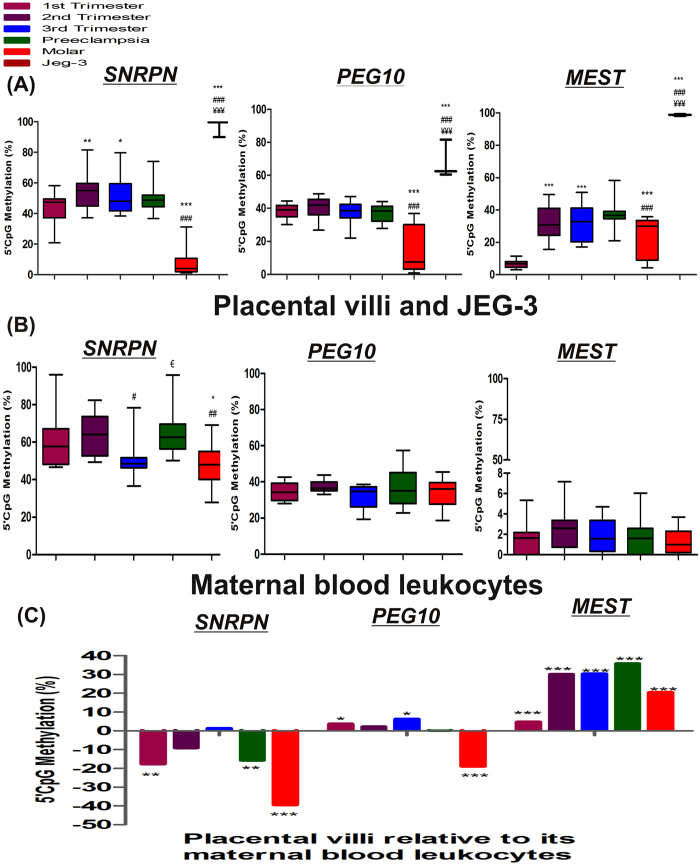
DNA methylation at DMRs of imprinting genes. (**A**) Box-and-whisker plot to show % CpG methylation among different placental villous samples and JEG-3 cells, (**B**) Box-and-whisker plot to show % CpG methylation among maternal blood leukocytes [*p < 0.05, **p < 0.01, ***p < 0.001 *vs* 1^st^ trimester, ^#^p < 0.05, ^##^p < 0.01, ^###^p < 0.001 *vs* 2^nd^ trimester, ^€^p < 0.05 *vs* 3^rd^ trimester and ^¥¥¥^p < 0.001 *vs* molar], and (C) % CpG methylation in villous samples in reference to their corresponding maternal blood leukocytes [*p < 0.05, **p < 0.01,***p < 0.001]. n = 30 per group.

Figure 2A–C demonstrates significant variation in CpG methylation pattern between different groups for *SNRPN*, *PEG10* and *MEST*. As shown in Fig. 3A, increase in CpG methylation at *SNRPN* DMR was observed with advancing normal gestation, increasing by 12% (p < 0.01) in second trimester and by 9.3% (p < 0.05) in third trimester, as compared to first trimester, while *MEST* DMR was found to be hypomethylated in first trimester with almost 5 fold lower methylation (p < 0.001) than in other normal gestational groups. However, *PEG10* DMR which is located within its promoter showed no significant difference in methylation levels among normal gestational placenta all being in the range of 37–39% average methylation. Development of preeclampsia was not observed to be related with any significant change in the methylation of DMRs of *SNRPN*, *PEG10* and *MEST*. However, similar to the reverse trend observed in the mRNA levels of these genes in molar villi and JEG-3 cells, a reverse trend was observed in the CpG methylation in these groups. *SNRPN* and *PEG10* DMRs were observed to have significantly lower methylation in molar villi relative to first trimester villi being reduced by 34.2% and 23.3% respectively, whereas JEG-3 cells presented higher methylation at promoter region of these genes raised by 53.7% and 30% respectively, relative to first trimester villi. However, *MEST* DMR was observed to show higher methylation both in molar villi and JEG-3 cells (3.4- and 15.2- fold, p < 0.001, respectively) relative to normal first trimester placenta, although the relative methylation pattern of molar villi with respect to JEG-3 cells was similar to that observed for *SNRPN* and *PEG10*.

In case of maternal blood samples *SNRPN* DMR was observed to show the lowest methylation (50.6 ± 11.3%, p < 0.05) in third trimester in reference to second trimester. Development of preeclampsia was associated with 1.3 fold (p < 0.05) increase in methylation of *SNRPN* in maternal blood relative to normal third trimester blood, while molar pregnancy was associated with 1.26 (p < 0.05) and 1.33 (p < 0.01) fold decrease in *SNRPN* methylation relative to normal first and second trimester blood respectively. In case of *PEG10* and *MEST* there was no significant change in CpG methylation among different groups in mothers, although being relatively higher for *PEG10* ranging from 32–38% while being less than 2.5% for *MEST* DMR (Fig. 3B).

On comparison of methylation levels of these genes in placental villi versus their relative maternal blood samples, lower methylation was observed in all placental groups except in third trimester villi group in case of *SNRPN* DMR. In case of *PEG10* and *MEST* DMR, all placental groups showed significantly higher methylation relative to their corresponding maternal blood groups, expect that relatively lower methylation for *PEG10* observed in case of molar placental group (Fig. 3C).

Correlating the DMR methylation of these genes with their respective mRNA expression levels among placental villi groups and JEG-3 cells predicted a strong correlation between these variables, Pearson correlation coefficient ‘r’ was estimated to be −0.93 (p < 0.05), −0.97 (p < 0.01) and −0.73 for *SNRPN*, *PEG10* and *MEST* respectively. Furthermore, Pearson correlation analysis between DMR methylation of these genes was also detected to be highly significant among these groups ‘r’ being 0.99 (p < 0.001), 0.83 (p < 0.05) and 0.82 (p < 0.05) for *SNRPN* vs *PEG10*, *SNRPN* vs *MEST* and *PEG10* vs *MEST* respectively.

### DMRs/promoters of imprinting genes show occupation of histone3 lysine trimethylations

**Figure 4:**
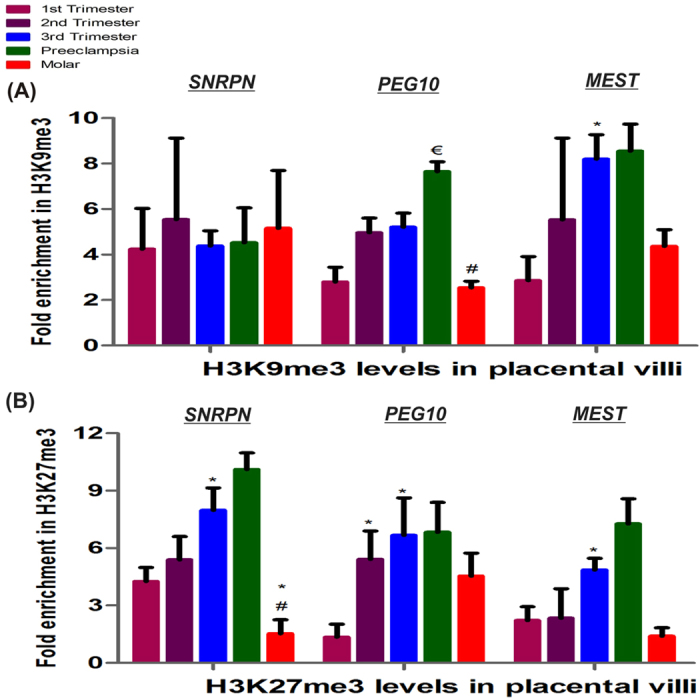
Quantification of histone trimethylation at DMRs of imprinting genes among placental villous groups. Fold enrichment relative to non-specific IgG acting as negative control and normalized with input DNA in (**A**) H3K9me3 and (**B**) H3K27me3. The data is presented as mean of the observed fold change ± SEM; n = 4 per group. *p < 0.05 *vs* 1^st^ trimester, ^#^p < 0.05 *vs* 2^nd^ trimester, ^€^p < 0.05 *vs* 3^rd^ trimester.

Transcription inhibiting histone modifications like histone trimethylation at lysine 9 and 27 (H3K9/K27me3), were quantified at *SNRPN*, *PEG10* and *MEST* DMRs/promoters in normal placental villi groups as well as preeclampsia and molar villi groups via ChIP assay relative to non-specific antibody as background in each placental villi group as shown in Fig. 4A and B. ChIP analysis detected almost equal fold enrichment in H3K9me3 level at *SNRPN* DMR among different categories with no significant difference observed. H3K9me3 at *PEG10* DMR, was found to be nonsignificantly increased in midgestation and term placenta, relative to first trimester placenta. ChIP analysis detected higher promoter occupancy by H3K9me3 in *MEST*, in third trimester relative to first trimester being raised by 2.8 fold (p < 0.05) (Fig. 4A).

A significant increase in fold enrichment of H3K27me3 was observed at DMRs of all three imprinting genes with advancing normal gestation which was found to be raised by 1.7-, 4.9- and 2.2- fold (p < 0.05) in third trimester relative to first trimester. Development of preeclampsia was observed to be associated with a significant increase of 1.5 fold (p < 0.05) in H3K9me3 level at *PEG10* DMR and some non-significant increase in levels of H3K27me3 at *SNRPN* and *MEST* DMRs. Furthermore, molar placental villi showed lower levels of H3K9me3 at *PEG10* DMR (1.9 fold, p < 0.05) and H3K27me3 at *SNRPN* and *MEST* (2.7 fold, p < 0.05 and 1.5 fold respectively) (Fig. 4B).

Multiple regression analysis performed to analyze the effect of epigenetic regulation via DNA methylation as well as H3K9/27me3 on mRNA expression of imprinting genes within placental villi groups, predicted higher regression coefficient “r^2^”, which was estimated to be 0.99 (p < 0.05) for both *SNRPN* and *PEG10*, while r^2^ was 0.84 for *MEST* (Table 1). Comparing individual variables with mRNA expression of these genes using Pearson correlation analysis confirmed strong correlation between mRNA expression of *SNRPN* with its H3K27me3 levels (r = −0.89, p < 0.05), *PEG10* with its H3K9me3 (r = −0.73) and *MEST* with both its H3K9me3 (r = −0.87) and H3K27me3 (r = 0.88, p < 0.05).

### Expression of *SNRPN*, *PEG10* and *MEST* in relation to the methylation at their DMRs in placental cell lines

**Figure 5:**
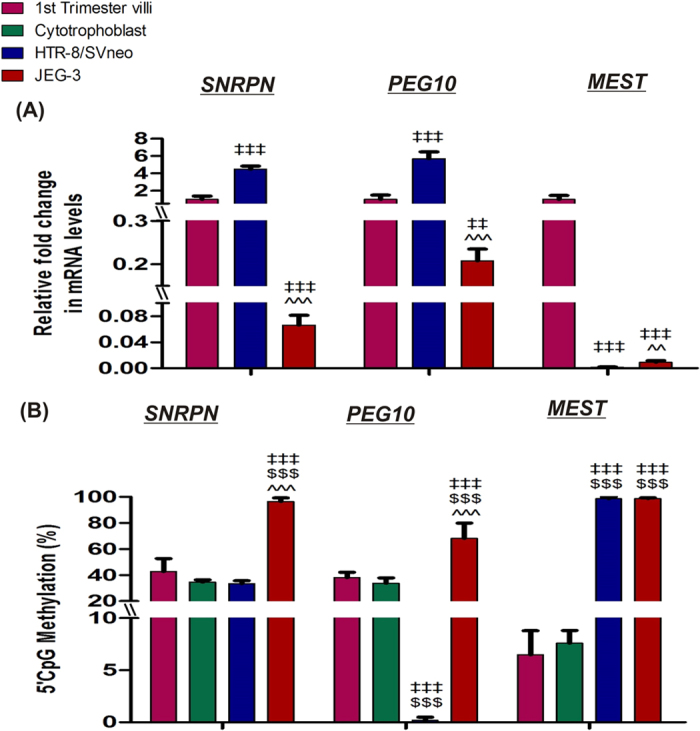
Regulation of differential mRNA expression via DNA methylation in placental cell lines, isolated cytotrophoblasts and normal first trimester placental villi. (**A**) qRT-PCR analysis of candidate tumor suppressor genes. (**B**) MS-HRM analysis of candidate tumor suppressor genes. ^‡‡^p < 0.01, ^‡‡‡^p < 0.001 *vs* first trimester villi, ^$$$^p < 0.001 *vs* isolated cytotrophoblasts and ^^^^p < 0.01, ^^^^^p < 0.001 *vs* HTR-8/SVneo. The data is presented as mean of observed percentage methylation ± SEM of three experiments for cell lines, isolated cytotrophoblasts and for 30 patients in first trimester placental villi group.

Comparison of the relative mRNA expression levels of *SNRPN, PEG10* and *MEST* in JEG-3 and HTR-8/SVneo cell lines and normal first trimester villous tissue (Fig. 5A), revealed the lowest expression of *SNRPN* and *PEG10* in JEG-3 cells being 15 fold (p < 0.001) and 5-fold (p < 0.01) lower than normal first trimester villi, while the expression of these genes was observed to be the highest in HTR-8/SVneo cells (raised by 4.4- and 5.7- fold, respectively, p < 0.001). However, there was almost negligible expression of *MEST* in HTR-8/SVneo and JEG-3 cells relative to first trimester villi (p < 0.001). Estimation of the DMR methylation percentage for *SNRPN. PEG10* and *MEST* revealed inverse trend in methylation percentage in JEG-3, HTR-8/SV/neo cells and first trimester villi as that observed for their mRNA expression suggesting DNA methylation mediated regulation of their mRNA expression (Fig. 5B). In addition the methylation status of these genes in isolated first trimester cytotrophoblasts was observed to be almost similar to that observed in first trimester villi. MS-HRM analysis revealed hypermethylation at *SNRPN* DMR (96.5% ± 2.67%), while it was moderately methylated in HTR-8/SVneo cells, cytotrophoblasts and first trimester villi (33.5 ± 2.13, 34.6 ± 1.7 and 42.7 ± 10%, respectively). *PEG10* DMR was detected to be almost unmethylated in HTR-8/SVneo cells (0.18 ± 0.3%), hypermethylated in JEG-3 cells (68.2 ± 11.6%) and moderately methylated in normal first trimester villi (38 ± 4%) and isolated first trimester cytotrophoblasts (33.8 ± 4). *MEST* DMR was observed to be equally hypermethylated in JEG-3 and HTR-8/SVneo with almost 98% methylation, while normal first trimester placenta and cytotrophoblasts were found to be hypomethylated with 6.4 ± 2.3 and 7.5 ± 1.2% methylation, respectively.

### Folic acid levels in placental villi groups

**Figure 6:**
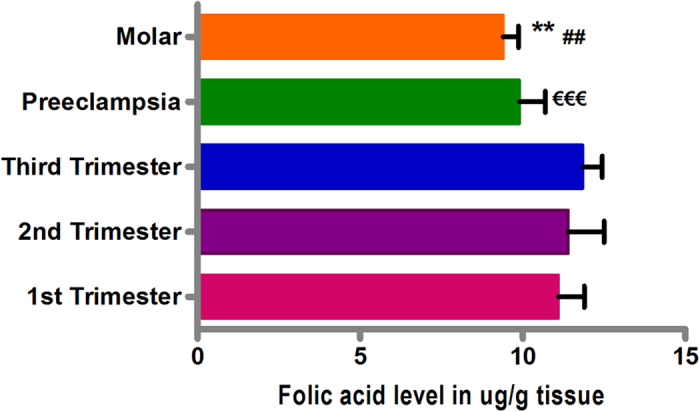
Relative Folic acid levels among different placental villi samples categories. Folate levels in ug/g of villous tissue in normal 1^st^, 2^nd^ and 3^rd^ trimester, preeclampsia and molar villi samples. The data are expressed as mean value ± SEM. **p < 0.01, ^##^p < 0.01 w.r.t second trimester and ^€€€^p < 0.001 w.r.t third trimester.

Estimation of folic acid levels in placental villous samples from different study groups revealed significantly decreased folate levels in preeclamptic placental villi (9.89 μg/g tissue, p < 0.001) and molar villi (9.39 μg/g tissue, p < 0.01), in reference to their respective control groups (Fig. 6).

### Effect of folic acid supplementation on mRNA expression and DNA methylation of imprinting genes

**Figure 7:**
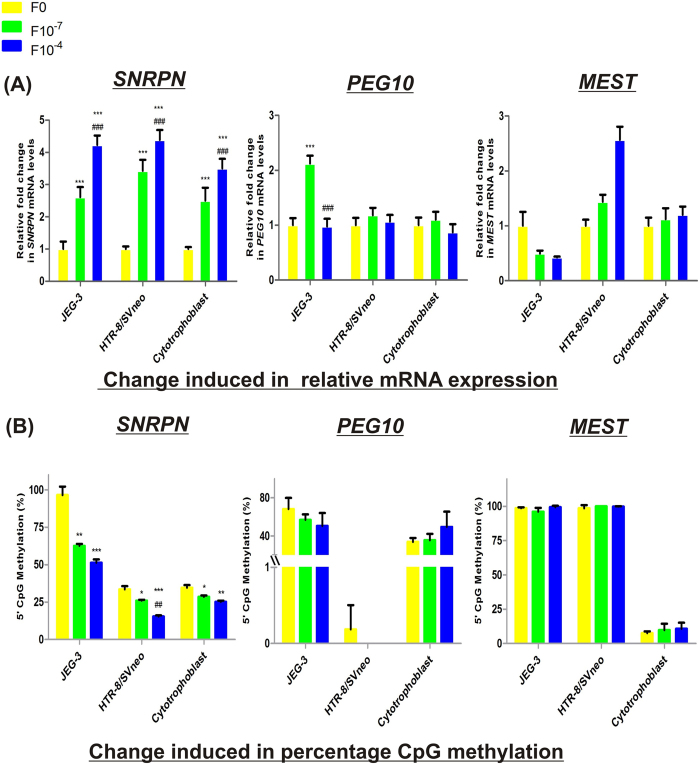
Effect of folic acid supplementation on imprinting gene regulation in placental cell lines and isolated cytotrophoblasts. (**A**) Effect of folic acid supplementation on relative mRNA expression of imprinting genes. (**B**) Effect of folic acid supplementation on percentage CpG methylation at DMRs of imprinting genes. F0-without folic acid supplementation, F10^−7^ and F10^−4^ - folic acid supplementation at the concentration of 10^−7^ and 10^−4^ M respectively. The data is presented as mean ± SEM, of three experiments. *p < 0.05, **p < 0.01, ***p < 0.001 w.r.t the respective F0 control and ^##^p < 0.01, ^###^p < 0.001 w.r.t the respective F10^−7^ treated cells.

In order to find the possible effect of folic acid supplementation at two different concentrations i.e. 10^−7^M and 10^−4^ M [which represents the concentrations in physiological range (400–600 μg/day) and a much higher concentration than physiological range respectively] on the mRNA levels of imprinting genes in placenta and the contribution of altered DNA methylation in it, we analyzed the mRNA expression and DMR/promoter methylation of *SNRPN*, *PEG10* and *MEST* under these conditions. Folic acid supplementation strongly increased the expression of *SNRPN* at both the concentrations. In JEG-3, HTR-8/SVneo cells and cytotrophoblasts it raised the expression by 2.6-, 3.4- and 2.5-fold at 10^−7^ M folic acid supplementation with respect to their relative control cells (p < 0.001) and then further by 1.6-, 1.3- and 1.4- fold (p < 0.01) at 10^−4^ M, relative to their respective 10^−7^ M folic acid supplemented cells. However, folic acid was able to induce the mRNA expression of *PEG10* by 2.12 fold (p < 0.001), only in case of JEG-3 cells at 10^−7^ M concentration, relative to control cells. Folic acid showed a differential cell line specific response in the mRNA expression of *MEST,* decreasing it by almost 2 fold (p < 0.001) in JEG-3 cells and increasing by 1.4 fold (p < 0.01) in HTR-8/SVneo cells at 10^−7^ M folic acid concentration and then further by 2.5 fold (p < 0.001) at 10^−4^ M folic acid concentration relative to untreated cells (Fig. 7A). Folate supplementation induced no significant change in the expression of MEST in first trimester cytotrophoblasts.

Analyzing the effect of folic acid supplementation on CpG methylation of these imprinting genes revealed a significant dose dependent effect on *SNRPN* methylation in JEG-3, HTR-8/SVneo cell lines and cytotrophoblasts, decreasing DMR methylation by 1.5- (p < 0.01), 1.2- (p < 0.05) and 1.2- (p < 0.05) fold, respectively, at 10^−7^ M folic acid concentration with respect to untreated cells. Similarly, it decreased by 1.8-, 2.2- (p < 0.001) and 1.4- (p < 0.01) fold, respectively, at 10^−4^ M folic acid concentration with respect to untreated cells. However, folic acid supplementation was not observed to induce any significant change in methylation at DMRs of *PEG10* and *MEST* (Fig. 7B). Pearson correlation analysis upon folic acid supplementation revealed high inverse correlation between the change induced in mRNA expression and DNA methylation of *SNRPN* (r = −0.96).

### Fetal originated imprinting gene DMRs in maternal plasma as epigenetic DNA markers

**Figure 8:**
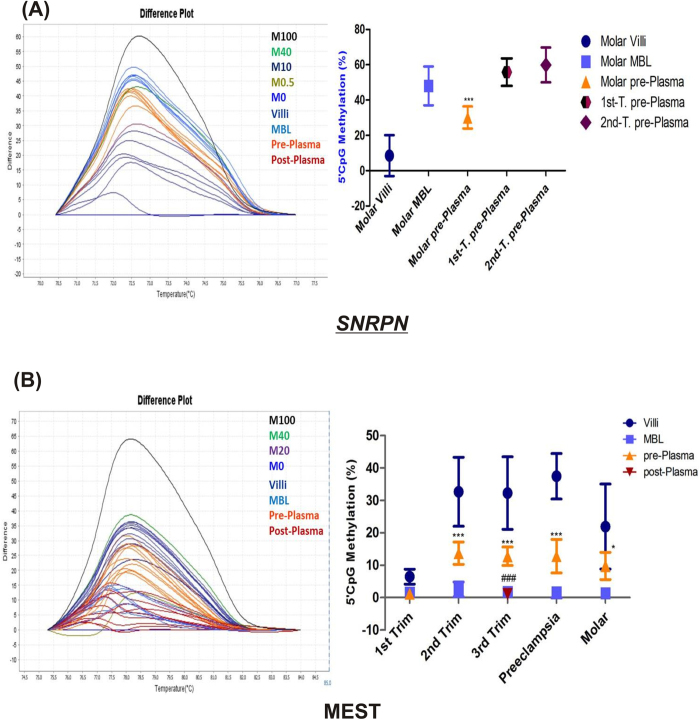
Evaluation of *SNRPN* and *MEST* as a fetal DNA epigenetic marker. (**A**) *SNRPN* and (**B**) *MEST*, each figure represents a difference plot showing methylation standards in different colors (M100%–M0%, which stands for DNA methylation standards with 100 to 0% methylation) normalized to the 0%-methylated standard DNA and few representative samples of each group and a graphical representation of % CpG methylation detected within placental villous samples, maternal blood leukocytes (MBL), maternal plasma samples obtained pre- obstetric procedure (pre-Plasma) and post-delivery (post-Plasma, only in case of third trimester group) from each group. The data is presented as mean percentage methylation ± SEM, n = 30 per group. *p < 0.05, ***p < 0.001 w.r.t the respective maternal blood leukocytes and ^###^p < 0.001 w.r.t the pre-plasma.

In order to analyze the potential of DMRs of imprinting genes to act as fetal DNA epigenetic marker in maternal blood we analyzed the methylation of DMRs, in cell free circulating DNA in maternal plasma which is a combination of maternal DNA derived from maternal leukocytes as well as fetal DNA derived from placenta. In this study, *SNRPN* and *MEST* DMRs were observed to show significantly different methylation percentage in placental villi groups relative to their corresponding maternal blood DNA. *SNRPN* DMR was observed to be show lower methylation in molar placenta when compared to molar blood samples, based on this difference we analyzed *SNRPN* methylation in molar maternal plasma samples (Fig. 8A), where a 18% (p < 0.001), decrease in *SNRPN* DMR methylation, relative to that in molar blood DNA was observed. This shows the presence of hypomethylated fetal *SNRPN* in maternal plasma, highlighting the potential of *SNRPN* to act as an epigenetic marker specific for molar pregnancy. The potential of *MEST* DMR to act as a fetal epigenetic marker in maternal plasma was analyzed in all groups (Fig. 8B), since there was a clear and significant difference in *MEST* DMR methylation between placental DNA and maternal blood DNA in all study groups. *MEST* DMR methylation was found to be significantly higher relative to the respective maternal blood leukocyte DNA in normal second and third trimester, preeclampsia and molar groups (p < 0.001, p < 0.05). The average *MEST* DMR methylation in plasma samples being 14 ± 3.4% in second trimester, 13 ± 2.8% in third trimester, 13 ± 5.1% in preeclampsia and 10 ± 4.2% in molar, thus showing the potential of *MEST* to act as fetal DNA epigenetic marker during normal gestation after midgestation as well as in placental disorders. The absence of higher methylation at *MEST* DMR in first trimester maternal plasma and the observed significantly higher methylation at *MEST* DMR in molar maternal plasma, suggests the use of *MEST* DMR as a marker specific to diagnose molar pregnancy in reference to normal early pregnancy.

## Discussion

This is the first study to show association of abnormal DNA and histone methylation at DMRs of *SNRPN*, *PEG10* and *MEST* with the development of placental disorders. The most prominent physiological resemblances between placentation and cancer development is the proliferation and invasion of cells into surrounding tissue. Such resemblances are attributed to similar expression of various growth factors and tumor like methylome in placenta^3^,^4^. However, unlike cancer development the proliferation and invasion processes exhibited by placental trophoblasts are tightly regulated during normal placentation. Any variation, lower or higher than the physiological level leads to placental disorders like preeclampsia and hydatiform mole, respectively. In this context, we intended to find the role of three paternally expressed genes: *SNRPN*, *PEG10* and *MEST*, with known function in placental development and carcinogenesis^38^,^39^,^40^. These genes are involved in mediating cell proliferation, invasion and growth, thus, highlighting their potential in tumor formation. The biological importance of the regulated expression of these genes in human development can be estimated by the fact that any abnormal change within the chromosomal regions harboring these genes have been associated with various syndromes like Angelman syndrome or Prader-Willi syndrome^41^,^42^, Silver–Russell syndrome (SRS)^31^ and cancer development^43^.

Based on this background knowledge, we hypothesized the possible role of epigenetic deregulation at DMRs of *SNRPN, PEG10* and *MEST* in development of placental disorders. This was studied in reference to the epigenetic regulation of these genes during normal placentation. Dynamic nature of placental gene expression has been referred to be an adaptive mechanism which allows placenta to coordinate with changing gestational conditions^44^.

In our study, the mRNA expressions of *SNRPN*, *PEG10* and *MEST* were observed to decrease with advancing gestation in placental tissue and seemed to be regulated by increased methylation at their DMRs as well as H3K27me3 level and additionally increased H3K9me3 levels for *MEST*. Based on the importance of these genes in tumor growth, the observed high expression of these genes in early pregnancy suggests the possible role of these genes in high tumorous behavior associated with early gestational placenta. In light of these studies and our results we suggest the role of both DNA and histone methylation at DMRs of these genes in regulating normal pregnancy. Studies have also reported a mechanistic relationship between DNA methylation and histone modifications present at DMRs of *SNRPN*^45^, *PEG10*^46^ and *MEST*^47^, suggesting the role of DNA methylation in acquisition or preservation of histone methylation at these regions^48^. Differential mRNA expression of these genes had not been estimated earlier during advancing gestation in human placenta. Although, the importance of these genes is supported by the reported methylation defects at DMRs of these imprinting genes associated with assisted reproductive technology^25^,^40^,^49^,^50^,^51^.

The fact that molar pregnancy arises due to fusion of a sperm with an ovum lacking maternal chromosome^52^, supports the observed higher expression of these paternally expressed genes in molar villi, mediated by decreased methylation at their DMRs, relative to normal early gestation placenta. Further, it was also observed to be associated with decreased H3K27me3 level at *SNRPN* DMR and H3K9me3 level at *PEG10* DMR. This observed epigenetic dysregulation and upregulation of gene expression in molar villi might be contributing to enhanced cellular proliferation and invasion of molar tissue, as these genes are known to show similar aberrant changes in various tumors like Wilms and germ cell tumors^53^,^54^. However, in JEG-3 cells minimal expression of these genes mediated by abnormally higher methylation at their DMRs, which suggests the possible role of aberrant hypermethylation at these DMRs in development of choriocarcinoma. Previously, deregulation of *IGF2* and *H19* genes has also been observed in GTDs^55^,^56^. This observed difference in molar placenta and JEG-3 cells can also be attributed to the fact that choriocarcinoma cell lines exhibit highly complex chromosomal aberrations^57^.

Whereas, development of preeclampsia, which involves lower growth and invasive potential of placental trophoblasts, was associated with down regulation of these placental growth and invasion promoting genes^21^. This decreased expression of *SNRPN* and *MEST* seemed to be regulated mainly by increased H3K27me3 levels in preeclamptic villi relative to normal third trimester villi. *MEST* expression was also found to be abnormally lower in preeclamptic maternal blood suggesting the role of overall decrease in *MEST* levels in development of preeclampsia. Decreased expression of *MEST* as observed in our study, might be contributing to poor angiogenesis observed in preeclampsia, as supported by the role of *MEST* in trophoblastic angiogenesis^27^. Supporting this phenotype lower mRNA levels of *MEST* have also been reported in intrauterine growth restricted placentas^58^.

Within in all studied placental villi groups a strong transcriptional regulation of these genes via CpG methylation and histone modifications is supported by their observed higher multiple regression coefficient value (r^2^ = 0.99, p < 0.05, in case of *SNRPN* and *PEG10*, while r^2^ = 0.84 in case of *MEST*). Moreover, significantly high positive correlation between the DNA methylation levels at DMRs of these three genes within these placental villi groups, suggests some similar mechanism regulating their DNA methylation within placenta.

Analyzing the effect of DNA methylation at DMRs of *SNRPN, PEG10* and *MEST* in JEG-3 and HTR-8/SVneo cells in reference to normal first trimester villi revealed hypermethylation mediated least expression in JEG-3 cells in case of *SNRPN* and *PEG10*, whereas *MEST* DMR was observed to be hypermethylated both in JEG-3 and HTR-8/SVneo cells with corresponding lower expression relative to normal first trimester villi. Thus, suggesting strong transcriptional regulation of these genes in these cell lines.

In general, folic acid supplementation is usually recommended priconceptionally during early pregnancy and even till late midgestation in some cases. Therefore, analyzing the effect of folic acid supplementation on the mRNA expression of these placental genes is of predominant significance. Furthermore, folic acid is also used as a therapy to restore the methylation and expression levels of various genes^59^. In this context, our study revealed decreased methylation at DMR of *SNRPN* upon folic acid supplementation in a dose dependent manner, supported by their strong negative correlation (r > −0.87) and correlated with the corresponding increase in mRNA expression of *SNRPN* (r > −0.94). Such inverse relation between folate level and DNA methylation was also found by Hoyo *et al*.^60^ for *H19*^60^. The decreased methylation of *SNRPN*, upon folic acid supplementation might be due to decreased expression of *DNMT3A*, which was also found to be down-regulated by folic acid supplementation in our study (unpublished data), as *DNMT3A* is specifically involved in methylation of imprinting genes^61^. It is also supported by the high correlation observed in our study, between decreasing methylation of *SNRPN* and decreasing expression of *DNMT3A* (r = 0.99, p < 0.05). Such increased expression upon folic acid treatment in case of *PEG10*, was observed only in choriocarcinomic JEG-3 cells, at lower folic acid supplementation while no such change was induced in extravillous HTR-8/SVneo cell line. Overexpression of *PEG10* has earlier been correlated with various cancers such as B-cell lymphocytic leukemia and hepatocellular carcinoma^39^. Therefore, folic acid mediated increase in *PEG10* expression, might be promoting malignancy like phenotype of placental development. Our results for *MEST* mRNA expression showed a differential cell line specific response, upon folic acid supplementation, with inhibitory effect in JEG-3 cells and activating effect in HTR-8/SVneo cells. This supports the earlier study showing the cell line specific effect of folic acid supplementation^62^. Folic acid has also been reported to induce such differential expression of genes in lymphoblastoid cells^63^. Previous studies have reported increased methylation at DMR of IGF2 in children, upon periconceptional supplementation of maternal folic acid^64^, whereas, supplementation of folic acid after 12 weeks of gestation has been correlated with increased level of methylation at IGF2 and decreased methylation at PEG3 and LINE1^65^. Thus, our results and these studies emphasize the folate induced regulation of gene expression and DNA methylation in a manner specific to genes and particular cell type.

The significance of fetal DNA epigenetic markers has been highlighted by the recent discovery of a few such markers and their utility in non-invasive prenatal diagnosis and early diagnosis of pregnancy related disorders^66^,^67^. Based on this knowledge, we designed the other part of our study aimed to explore the potential of the DMRs of these selected genes as fetal DNA epigenetic marker. For this purpose, we analyzed the DMRs which were observed to be differentially methylated between maternal DNA and placental DNA in our study. Therefore, we selected *MEST* and *SNRPN* DMRs based on our data and analyzed these DMRs in maternal plasma to search for methylation pattern specific to fetal (placental) DNA. Previously, the difference among maternal and fetal methylation levels at ICRs of imprinting regions had been utilized for the development of fetal specific epigenetic markers in an allele specific pattern. The ICRs of Igf2 and H19 genes were analyzed in maternal plasma in order to search for fetal specific methylation pattern^68^. However, this approach required further genotypic analysis of differentially methylated region via biallelic polymorphism for confirming the results. On the other hand, according to our procedure such confirmation is not required, as our approach is not specific to any allele, instead we searched for the methylation pattern mimicking the pattern observed for fetal (placental) DNA, thus, reflecting the presence of fetal DNA in maternal plasma. Therefore, based on our results the presence of fetal DNA can be determined in maternal plasma via a single step and simpler test.

In our study we have observed *MEST* as a potential fetal DNA epigenetic marker both in normal as well as in complicated pregnancies. Although, the observed methylation level of *MEST* DMR/promoter was less than 20% in different groups but it was easily detectable in the background of unmethylated MEST DMR in maternal blood cells. Moreover, *MEST* DMR was found to be higher methylated in maternal plasma in all groups, except in the first trimester plasma where it was found to be hypomethylated in comparison to maternal plasma of molar pregnancies, thus, giving an additional advantage to *MEST* DMR to be used as a diagnosis marker for molar pregnancies. In case of *SNRPN* DMR lower methylation was observed in placental villi in comparison to maternal blood, specifically in molar group, resulting in a drop of methylation in maternal plasma. Thus, suggesting *SNRPN* to be a potential epigenetic marker specific to molar pregnancy. Similar pattern of lower methylation has previously been reported for *SERPINB5 (maspin*) promoter in maternal plasma and the raised concentration of its unmethylated sequences has been correlated with preeclamptic pregnancies in reference to normal term pregnancies^67^.

There are few limitations in the present study. Firstly, the sample size is small in order to fully validate the observed fetal DNA epigenetic markers. However, this is a preliminary study which screened these DMRs for their potential to act as fetal DNA epigenetic marker in different groups and thus, can be further validated in larger population. Secondly, this study has not analyzed the allele specific expression of these genes which are known to be involved in imprinting. This study intended to compare the relative expression and the total amount of DNA and histone methylation present at the DMRs also known as their promoter regions, of these genes irrespective of its origin, between different physiological and pathological groups. Therefore, this study can provide a sufficient insight to further analyze the allele specific expression of these genes in different groups.

In summary, these data present the first evidence supporting the importance of tight regulation of both DNA methylation and histone trimethylation at DMRs of *SNRPN*, *PEG10* and *MEST* during physiological pregnancy and suggests the dysregulation of these modifications as contributing factor for development of gestational trophoblastic diseases and preeclampsia. Our data also suggests the role of higher expression of these genes in enhancing the malignant phenotype of placenta during early pregnancy and the possible gene specific effect of folic acid supplementation which may be further enhancing the malignant phenotype of placenta. Moreover, our study highlights the potential of *MEST* and *SNRPN* DMRs to act as fetal DNA epigenetic marker for their use in prenatal diagnosis or early diagnosis of placental pathologies, which however, needs to be further validated in larger population and in early gestational pregnancies.

## Methods

### Study approval

Consent was taken in writing from all pregnant women included in this study after clearly informing them about the study. Patient samples were collected and processed according to the relevant instructions and guidelines. The study had been approved by Institute Ethics Committee (IEC) of Postgraduate Institute of Medical Education and Research (PGIMER). All the experimental work was done at PGIMER after approval of the plan of work from institutional Dean Doctoral Committee (DDC).

### Design of the study and collection of biological samples

Pregnant women with normal gestation in three different trimesters of pregnancy (first, second and third trimester, n = 30 in each group) and two placental disorders: preeclampsia (n = 30) and hydatidiform mole (n = 15) were recruited in this study from department of Obstetrics and Gynecology of PGIMER, India. Pregnant women were included into different categories according to set criteria. The three normal gestation groups first-, second- and third-trimester included pregnant women of 6–11 weeks, 16–20 weeks and 37–40 weeks pregnancy respectively. Pregnant women diagnosed with clinical symptoms of 140/90 mm Hg systolic/diastolic pressure, proteinuria >300 mg in 24 hr were included in preeclamptic group while hydatidiform mole group included pregnant women in early gestation diagnosed for molar pregnancy by ultrasonography and confirmed by histopathology. Pregnant women with any medical complication like (gestational diabetes or diabetes mellitus, hypertension, thyroid malfunctions and any reported infection), placenta previa, fetal anomaly, known genetic disorder in previous children or fetal malformations, oligohydramnios, hydramnios, preterm labor, premature rupture of the membranes during the present pregnancy, were excluded from our normal trimester groups. The demographic characteristics of pregnant women included in this study are given in Table 2. Fresh placental tissues were obtained from normal gestational as well as complicated pregnancies either after caesarean delivery in case of third trimester or preeclamptic pregnancies or termination of pregnancy in case of first and second trimester or molar pregnancies. We took almost 5 mm^3^ of chorionic villi section beneath 5 mm of the outer layer of the placenta. This position was kept almost constant for different placentae and was selected near umbilical cord insertion region. Chorionic villi were dissected and separated from amniochorionic membranes, blood vessels and maternal tissue under microscope, followed by removal of fetal and maternal blood by rinsing with phosphate buffered saline. Tissue samples were also routinely for their histopathology by hematoxylin and eosin staining, in order to check the accuracy of sample collection and to check the morphology of placental villi obtained from different trimester. From each pregnant woman 10 mL of maternal peripheral blood was withdrawn before any obstetric procedure. These maternal blood samples were used for the isolation of plasma and maternal blood leukocytes^69^. In addition, maternal blood samples were also collected after 24 hrs of delivery from pregnant women with normal full term pregnancy, later used for isolation of plasma DNA. Placental tissue samples, isolated maternal blood leukocytes and plasma samples were then stored at −80 °C till isolation of DNA and RNA.

### *In-vitro* Cell culture based studies

*In-vitro* studies were done using two adherent placental cell lines *viz* a choriocarcinoma cell line (JEG-3) and transformed extravillous trophoblast cell line (HTR-8/SVneo) (Source: American Type Culture Collection -ATCC). JEG-3 cells were maintained in DMEM-HG (Dulbecco’s Modified Eagle’s Medium-High Glucose), with 4500 mg/L glucose and HTR-8/SVneo cell line were maintained in RPMI-1640 medium, supplemented with L-glutamine and sodium bicarbonate (3.7- and 2-g/L respectively), HEPES (25 mM) and FBS (10%). Both the cell lines were used to study the expression of *SNRPN*, *PEG10* and *MEST* and their regulation via DNA methylation. Additionally, these cell lines were also used to study the effect of folic acid supplementation on the expression of these imprinting genes and their DNA methylation under physiological concentration (10^−7^ M) and higher than physiological folate level (10^−4^ M). Folic acid was dissolved in 1 M NaOH and cells were treated for 48 hrs.

### Quantitative mRNA estimation

For mRNA quantification studies, RNA was extracted from placental villous samples, maternal blood leukocytes and two placental cell lines (JEG-3 and HTR/SVneo) using TRIzol (Ambion, Life Technologies Corporation, California). One μg of isolated RNA isolated was used in reverse transcription to obtain cDNA using RevertAidTM M-MuLV-RT kit (MBI Fermentas, Life Sciences, USA). Endpoint qRT-PCR (quantitative reverse transcriptase polymerase chain reaction) was carried in Applied Biosystems real time PCR (Life Technologies Corporation, California) on reaction mixture prepared for each sample by mixing SYBR Green master mix (5 μL) with 60 ng of obtained first-strand cDNA template (1 μl), 500 nM of gene specific primers, 1.5 mM MgCl_2_ in a reaction volume of 10 μL. PCR products were amplified using gene specific primers. mRNA expression was normalized to the GAPDH (glyceraldehyde-3-phosphate dehydrogenase), for which the Ct values were observed to be constant across different trimesters. Relative fold changes in gene expression between different groups were calculated using the comparative threshold cycle or Ct method (ΔΔCT) method^70^. The primers used for mRNA estimation assay are shown in Table 3.

### Methylation-Sensitive High Resolution Melting (MS-HRM) analysis

Total genomic DNA isolated by DNA isolation kit (Real Genomics, Real Biotech Corporation, Taipei, Taiwan), from placental villi samples, maternal blood leukocytes, JEG-3 and HTR/SVneo cell lines, circulating DNA isolated by Miniprep DNA isolation kit (Bioserve, MD, USA), from maternal plasma were used to estimate the DMR/promoter region methylation percentage by MS-HRM (methylation sensitive high resolution melting analysis) as described previously^37^. The MS-HRM analysis is based on the method given by Wojdacz and Dobrovic^71^. It detects single nucleotide changes within an amplicon based on the observed changes in its thermodynamic characters using sensitive thermocycler and an intercalating dye. Bisulfite treatment was given to the isolated DNA using EZ DNA Methylation-Gold Kit (Zymo Research, USA), in order to induce nucleotide changes, converting unmethylated cytosines into uracil, which changes into thymidine after DNA replication. Methylation standards (i.e 100% and 0% methylation standards) were generated before any MS-HRM assay. For the 0% methylation standard we have used commercially available control DNA from Qiagen (EpiTect® Control DNA), while the enzymatic treatment of genomic DNA was used to synthesize fully methylated DNA (100% methylation standard) using M.SssI enzyme (CpG Methyltransferase, New England Biolabs, Beverly, MA, USA). These methylation standards were also subjected to bisulfite conversion. DNA standards of 0 and 100% methylation were mixed together in proper proportion to generate DNA standards of 0, 0.5, 5, 10, 20, 40, 60, 80 and 100%, methylated standards. In order to determine the bias in primers, each assay was standardized by amplifying 50% methylation DNA standard along with 100, 25 and 0% standards at different annealing temperatures and other conditions, followed by analysis of melt curves in order to select the condition which gives almost equal amplification of methylated and unmethylated peaks for 50% methylation standard. High resolution melting analysis was performed on Applied Biosystems® StepOnePlus™ Real-Time PCR using gene specific primers (Table 3). This was followed by analysis of raw melt curves in MS-HRM software version 3.0.1. from Applied Biosystems and then finally the exact percentage of methylation of unknown samples was estimated in reference to methylation standards by Polyfit interpolating function within program MatLab (The MathWorks, Inc., USA).

### Chromatin immunoprecipitation (ChIP) assay

Chromatin immunoprecipitation (ChIP) was performed on placental villi samples to estimate the levels of H3K9me3 and H3K27me3 at DMR/promoter region of genes as previously described^37^. Briefly, 25 mg of placental villi was fixed in 1.5% formaldehyde, followed by its disaggregation to a single cell suspension, which was subjected to sonication for shearing of chromatin. The sheared chromatin lysate was then divided into aliquots to be used for input DNA, immunoprecipitation with anti-trimethyl H3-K9/K27 Ab (Abcam, Cambridge, England, UK) and normal rabbit immunoglobulin G (IgG) in proper proportion. Phenol chloroform method was used for DNA isolation from input and immunoprecipitated complexes and then quantified by real time PCR using gene specific primers (Table 3). ChIP assays for each histone modification specific to a particular gene were done in triplicates. Data was normalized with input DNA and calculated relative to non-specific antibody as fold enrichment.

### Estimation of folate levels by microbiological assay

The folate levels within placental villi samples were estimated using *Lactobacillus casei* assay, as previously explained^72^. Briefly, 10% homogenate of villous tissue was prepared in folate extraction buffer containing 50 mM ascorbic acid. The homogenate was incubated for 10 min at 110 °C, followed by centrifugation and the treatment of separated supernatant with rat plasma conjugase, which hydrolyzes the polyglutamated folate to monoglutamated folate form. The free folate is utilized by *L. casei* for its growth, hence the levels of folic acid levels are determined by measuring bacterial growth. In each assay, the standards of known folic acid concentration were run along with the samples.

### Statistical methods

In our study the data from preeclampsia group has been compared to gestational age matched normal third trimester group, whereas molar group has been compared to normal first and second trimester group for their statistical analysis. For statistical analysis, of more than two groups we performed one-way analyses of variance ANOVA (α = 0.05) followed by Fisher post-hoc test. Student’s t-test was used to compare the significance of variance between two groups, using IBM SPSS statistical program (v.16, NY, USA) and GraphPad Prism (v.5.00.288) were used for statistical analysis. Further, the correlation between different parameters was calculated by Pearson’s correlation analysis. In addition, multiple regression analysis was carried out to study the effect of epigenetic regulatory mechanisms on the mRNA expression. Data were expressed as mean ± SEM and were considered statistically significant at p < 0.05.

## Additional Information

**How to cite this article**: Rahat, B. *et al*. Epigenetic modifications at DMRs of placental genes are subjected to variations in normal gestation, pathological conditions and folate supplementation. *Sci. Rep.* **7**, 40774; doi: 10.1038/srep40774 (2017).

**Publisher's note:** Springer Nature remains neutral with regard to jurisdictional claims in published maps and institutional affiliations.

## Figures and Tables

**Table 1:** Pearson correlation and multiple regression analysis for *SNRPN*, *PEG10* and *MEST* in all five placental villous groups.

		Pearson Correlation analysis: r			Multiple regression analysis with mRNA expression as dependent variable: r^2^ (SEM)^#^
Gene		H3K9me3	H3K27me3	mRNA vs DNA methylation	
*SNRPN*	mRNA	0.12	−0.89*	−0.93*	0.99* (0.00029)
	DNA methylation	−0.2	0.75		
*PEG10*	mRNA	−0.73	−0.31	−0.97**	0.99*(0.0042)
	DNA methylation	0.54	0.1		
*MEST*	mRNA	−0.87	−0.88*	−0.75	0.84 (0.0047)
	DNA methylation	0.88*	0.63		

R = Pearson coefficient, r^2^ = regression determination coefficient, SEM = standard error of measurement, UD = undetermined due to unmethylated gene DMR/promoter. ^**#**^Equation for Prediction for each gene was “mRNA expression = β1H3K9me3 + β2H3K27me3 + β3DNA methylation + α”, where β1, β2, β3 are regression coefficients and α is intercept of test. *p < 0.05 and **p < 0.01.

**Table 2:** Demographic characteristics of pregnant women included in this study.

	First trimester	Second trimester	Third trimester	Preeclampsia	Molar
No of pregnant women included	30	30	30	30	15
Gestational age (weeks ± SD)	8.2 ± 1.4	18 ± 1.6	38 ± 0.8	35.3 ± 2.5	13.6 ± 2.9
Maternal age (years)	27.9 ± 3.4	27.6 ± 3.9	28 ± 3.5	26 ± 3.3	25.1 ± 2.6
Average Blood pressures systolic/diastolic mm Hg	120/80	120/80	120/80	>140/90	120/80
Delivery mode/Sample collection	Dilation and Curettage for medical termination of pregnancy.	Dilation and Curettage for medical termination of pregnancy.	Caesarean section	Caesarean section	Dilation and Curettage for medical termination of pregnancy

**Table 3:** Primers sequences and their parameters used in this study.

Gene/Assay	Forward primer 5′–3′	Reverse primer 5′–3′	Amplicon size bp/annealing temperature °C/No. of CpGs in amplicon*	Genbank accession number
*SNRPN* qRT-PCR	CGAATCTTCATTGGCACCTT	AGCAACACCAGACCCAAAAC	143/60	NM_003097.3
*PEG10* qRT-PCR	CAAGCCACCACCAGGTAGAT	GAGGCACAGGTTCAGCTTTC	101/62	NM_001040152.1
*MEST* qRT-PCR	TGGGCTTCATCAACTCCTTC	AGGACCTCTTTTGGGCATTT	112/62	NM_001253900.1
GAPDH qRT-PCR	CGACCACTTTGTCAAGCTCA	AGGGGTCTACATGGCAACTG	228/60-65	NM_001256799.2
*SNRPN* MS-HRM	GTTATCGGTATAGTTGATTTTGTT	AAACCTACCGCTACTACAAC	123/58/12	U41384.1
*PEG10* MS-HRM	GGTTTATAGTTTGCGTTTTTGGTAT	CGATCTCCACTAAATACTCC	176/58/16	NG_011340.1
*MEST* MS-HRM	GAAGGCGGTAGTATATGTTGGGT	CGACCTTCACCCTATTCCCAAAA	118/62/13	NG_009226.1
*SNRPN* qChIP-PCR	CCTCTGAACATTCCGGATCTG	AACGGAATTTGGGCCCTAAA	60/60	U41384.1
*PEG10* qChIP-PCR	CCGTCCGTCCTCGATTCTC	GCTAGAGGGAGTACGGGATTACC	108/62	NG_011340.1
*MEST* qChIP-PCR	CTCGTGCCCTTGGTGGTTA	GCCACGAGGGCCCTATG	57/62	NG_009226.1

*In case of only MS-HRM assays. **qRT-PCR:** quantitative Reverse transcriptase polymerase chain reaction. **MS-HRM:** Methylation- sensitive high resolution melting. **qChIP:** quantitative Chromatin immunoprecipitation assay.

## References

[b1] Ferretti C., Bruni L., Dangles-Marie V., Pecking A. P. & Bellet D. Molecular circuits shared by placental and cancer cells, and their implications in the proliferative, invasive and migratory capacities of trophoblasts. Hum Reprod Update 13, 121–141, doi: dml048 10.1093/humupd/dml048, doi: 10.1093/humupd/dml048(2013) J. Cell Biol (2007). 17068222

[b2] Soundararajan R. & Rao A. J. Trophoblast ‘pseudo-tumorigenesis’: significance and contributory factors. Reprod Biol Endocrinol 2, 15, doi: 10.1186/1477-7827-2-151477-7827-2-15 (2004). 15043753 PMC407853

[b3] D’Souza A. W. & Wagner G. P. Malignant cancer and invasive placentation: A case for positive pleiotropy between endometrial and malignancy phenotypes. Evol Med Public Health 2014, 136–145, doi: 10.1093/emph/eou022eou022 (2014). PMC4217742 25324490

[b4] Novakovic B. & Saffery R. Placental pseudo-malignancy from a DNA methylation perspective: unanswered questions and future directions. Front Genet. 4, 285, doi: 10.3389/fgene.2013.00285 (2013). 24368911 PMC3857887

[b5] Moore T. & Haig D. Genomic imprinting in mammalian development: a parental tug-of-war. Trends Genet. 7, 45–49, doi: 10.1016/0168-9525(91)90230-N (1991). 2035190

[b6] Miyoshi N., Barton S. C., Kaneda M., Hajkova P. & Surani M. A. The continuing quest to comprehend genomic imprinting. Cytogenet Genome Res 113, 6–11, doi: 10.1159/000090808 (2006). 16575156

[b7] Barton S. C., Surani M. A. & Norris M. L. Role of paternal and maternal genomes in mouse development. Nature 311, 374–376 (1984). 6482961 10.1038/311374a0

[b8] Wang X., Miller D. C., Harman R., Antczak D. F. & Clark A. G. Paternally expressed genes predominate in the placenta. Proc Natl Acad Sci USA 110, 10705–10710, doi: 10.1073/pnas.13089981101308998110 (2013). 23754418 PMC3696791

[b9] Morgan H. D., Santos F., Green K., Dean W. & Reik W. Epigenetic reprogramming in mammals. Hum Mol Genet. 14 Spec No 1, R47–58, doi: 14/suppl_1/R47 10.1093/hmg/ddi114 (2005). 15809273

[b10] Ferguson-Smith A. C. Genomic imprinting: the emergence of an epigenetic paradigm. Nat Rev Genet. 12, 565–575, doi: 10.1038/nrg3032nrg3032 (2011). 21765458

[b11] Butler M. G. Genomic imprinting disorders in humans: a mini-review. J Assist Reprod Genet. 26, 477–486, doi: 10.1007/s10815-009-9353-3 (2009). 19844787 PMC2788689

[b12] Eggermann T., Eggermann K. & Schonherr N. Growth retardation versus overgrowth: Silver-Russell syndrome is genetically opposite to Beckwith-Wiedemann syndrome. Trends Genet. 24, 195–204, doi: 10.1016/j.tig.2008.01.003S0168-9525(08)00054-1 (2008). 18329128

[b13] Devriendt K. Hydatidiform mole and triploidy: the role of genomic imprinting in placental development. Hum Reprod Update 11, 137–142, doi: 10.1093/humupd/dmh060 (2005). 15677707

[b14] Arnaud P. & Feil R. Epigenetic deregulation of genomic imprinting in human disorders and following assisted reproduction. Birth Defects Res C Embryo Today 75, 81–97, doi: 10.1002/bdrc.20039 (2005). 16035043

[b15] Feinberg A. P. The epigenetics of cancer etiology. Semin Cancer Biol. 14, 427–432, doi: S1044579X04000483 10.1016/j.semcancer.2004.06.005 (2004). 15489135

[b16] Oudejans C. B. . The parent-of-origin effect of 10q22 in pre-eclamptic females coincides with two regions clustered for genes with down-regulated expression in androgenetic placentas. Mol Hum Reprod. 10, 589–598, doi: 10.1093/molehr/gah080gah080 (2004). 15208369

[b17] Kanayama N. . Deficiency in p57Kip2 expression induces preeclampsia-like symptoms in mice. Mol Hum Reprod. 8, 1129–1135 (2002). 12468647 10.1093/molehr/8.12.1129

[b18] Georgiades P., Watkins M., Burton G. J. & Ferguson-Smith A. C. Roles for genomic imprinting and the zygotic genome in placental development. Proc Natl Acad Sci USA 98, 4522–4527, doi: 10.1073/pnas.081540898081540898 (2001). 11274372 PMC31867

[b19] Brosens J. J., Pijnenborg R. & Brosens I. A. The myometrial junctional zone spiral arteries in normal and abnormal pregnancies: a review of the literature. Am J Obstet Gynecol. 187, 1416–1423, doi: S0002937802004301 (2002). 12439541 10.1067/mob.2002.127305

[b20] Roberts C. T. IFPA Award in Placentology Lecture: Complicated interactions between genes and the environment in placentation, pregnancy outcome and long term health. Placenta 31 Suppl, S47–53, doi: 10.1016/j.placenta.2010.01.001S0143-4004(10)00002-0 (2010). 20096927

[b21] Dekker G., Robillard P. Y. & Roberts C. The etiology of preeclampsia: the role of the father. J Reprod Immunol. 89, 126–132, doi: 10.1016/j.jri.2010.12.010S0165-0378(11)00046-5 (2011). 21529966

[b22] Zaragoza M. V. . Parental origin and phenotype of triploidy in spontaneous abortions: predominance of diandry and association with the partial hydatidiform mole. Am J Hum Genet. 66, 1807–1820, doi: S0002-9297(07)63533-2 10.1086/302951 (2000). 10801385 PMC1378061

[b23] Redline R. W. & Abdul-Karim F. W. Pathology of gestational trophoblastic disease. Semin Oncol. 22, 96–108 (1995). 7740321

[b24] Cheung A. N., Zhang H. J., Xue W. C. & Siu M. K. Pathogenesis of choriocarcinoma: clinical, genetic and stem cell perspectives. Future Oncol. 5, 217–231, doi: 10.2217/14796694.5.2.217 (2009). 19284380

[b25] Chen S. . Assisted reproduction causes placental maldevelopment and dysfunction linked to reduced fetal weight in mice. Sci Rep. 5, 10596, doi: 10.1038/srep10596srep10596 (2015). 26085229 PMC4471727

[b26] Ono R. . Deletion of Peg10, an imprinted gene acquired from a retrotransposon, causes early embryonic lethality. Nat Genet. 38, 101–106, doi: 10.1038/ng1699 (2006). 16341224

[b27] Mayer W. . Expression of the imprinted genes MEST/Mest in human and murine placenta suggests a role in angiogenesis. Dev Dyn. 217, 1–10, doi: 10.1002/(SICI)1097-0177(200001)217:1<1::AID-DVDY1>3.0.CO;2-4 (2000). 10679925

[b28] Ozcelik T. . Small nuclear ribonucleoprotein polypeptide N (SNRPN), an expressed gene in the Prader-Willi syndrome critical region. Nat Genet. 2, 265–269, doi: 10.1038/ng1292-265 (1992). 1303277

[b29] Smallwood A. . Temporal regulation of the expression of syncytin (HERV-W), maternally imprinted PEG10, and SGCE in human placenta. Biol Reprod. 69, 286–293, doi: 10.1095/biolreprod.102.013078biolreprod.102.013078 (2003). 12620933

[b30] Frost J. M. & Moore G. E. The importance of imprinting in the human placenta. PLoS Genet. 6, e1001015, doi: 10.1371/journal.pgen.1001015 (2010). 20617174 PMC2895656

[b31] Kobayashi S. . Human PEG1/MEST, an imprinted gene on chromosome 7. Hum Mol Genet 6, 781–786, doi: dda101 (1997). 9158153 10.1093/hmg/6.5.781

[b32] Shemer R., Birger Y., Riggs A. D. & Razin A. Structure of the imprinted mouse Snrpn gene and establishment of its parental-specific methylation pattern. Proc Natl Acad Sci USA 94, 10267–10272 (1997). 9294199 10.1073/pnas.94.19.10267 PMC23351

[b33] Ono R. . Identification of a large novel imprinted gene cluster on mouse proximal chromosome 6. Genome Res. 13, 1696–1705, doi: 10.1101/gr.90680313/7/1696 (2003). 12840045 PMC403743

[b34] Lucifero D., Mann M. R., Bartolomei M. S. & Trasler J. M. Gene-specific timing and epigenetic memory in oocyte imprinting. Hum Mol Genet. 13, 839–849, doi: 10.1093/hmg/ddh104ddh104 (2004). 14998934

[b35] Figueiredo J. C. . Folic acid and risk of prostate cancer: results from a randomized clinical trial. J Natl Cancer Inst. 101, 432–435, doi: 10.1093/jnci/djp019 (2009). 19276452 PMC2657096

[b36] Brown J. E. . Predictors of red cell folate level in women attempting pregnancy. JAMA 277, 548–552 (1997). 9032161 10.1001/jama.1997.03540310046033

[b37] Rahat B., Hamid A., Ahmad Najar R., Bagga R. & Kaur J. Epigenetic mechanisms regulate placental c-myc and hTERT in normal and pathological pregnancies; c-myc as a novel fetal DNA epigenetic marker for pre-eclampsia. Mol Hum Reprod 20, 1026–1040, doi: 10.1093/molehr/gau053gau053 (2014). 25024139

[b38] Moon Y. S. . Imprinting and expression status of isoforms 1 and 2 of PEG1/MEST gene in uterine leiomyoma. Gynecol Obstet Invest. 70, 120–125, doi: 10.1159/000301555 (2010). 20339302

[b39] Lux H., Flammann H., Hafner M. & Lux A. Genetic and molecular analyses of PEG10 reveal new aspects of genomic organization, transcription and translation. PLoS One 5, e8686, doi: 10.1371/journal.pone.0008686 (2010). 20084274 PMC2800197

[b40] Mann M. R. . Selective loss of imprinting in the placenta following preimplantation development in culture. Development 131, 3727–3735, doi: 10.1242/dev.01241dev.01241 (2004). 15240554

[b41] Mascari M. J. . The frequency of uniparental disomy in Prader-Willi syndrome. Implications for molecular diagnosis. N Engl J Med. 326, 1599–1607, doi: 10.1056/NEJM199206113262404 (1992). 1584261 PMC7556354

[b42] Glenn C. C. . Gene structure, DNA methylation, and imprinted expression of the human SNRPN gene. Am J Hum Genet. 58, 335–346 (1996). 8571960 PMC1914536

[b43] Joyce J. A. & Schofield P. N. Genomic imprinting and cancer. Mol Pathol. 51, 185–190 (1998). 9893743 10.1136/mp.51.4.185 PMC395634

[b44] Radford E. J., Ferron S. R. & Ferguson-Smith A. C. Genomic imprinting as an adaptative model of developmental plasticity. FEBS Lett. 585, 2059–2066, doi: 10.1016/j.febslet.2011.05.063 (2011). 21672541

[b45] Horsthemke B. & Wagstaff J. Mechanisms of imprinting of the Prader-Willi/Angelman region. Am J Med Genet A 146A, 2041–2052, doi: 10.1002/ajmg.a.32364 (2008). 18627066

[b46] Monk D. . Comparative analysis of human chromosome 7q21 and mouse proximal chromosome 6 reveals a placental-specific imprinted gene, TFPI2/Tfpi2, which requires EHMT2 and EED for allelic-silencing. Genome Res. 18, 1270–1281, doi: 10.1101/gr.077115.108gr.077115.108 (2008). 18480470 PMC2493428

[b47] Skaar D. A. . The human imprintome: regulatory mechanisms, methods of ascertainment, and roles in disease susceptibility. ILAR J 53, 341–358, doi: 10.1093/ilar.53.3-4.341ilar.53.3-4.341 (2012). 23744971 PMC3683658

[b48] Henckel A. . Histone methylation is mechanistically linked to DNA methylation at imprinting control regions in mammals. Hum Mol Genet. 18, 3375–3383, doi: 10.1093/hmg/ddp277 (2009). 19515852

[b49] Kagami M., Nagai T., Fukami M., Yamazawa K. & Ogata T. Silver-Russell syndrome in a girl born after *in vitro* fertilization: partial hypermethylation at the differentially methylated region of PEG1/MEST. J Assist Reprod Genet. 24, 131–136, doi: 10.1007/s10815-006-9096-3 (2007). 17450433 PMC3455069

[b50] Suzuki J. Jr. . *In vitro* culture and somatic cell nuclear transfer affect imprinting of SNRPN gene in pre- and post-implantation stages of development in cattle. BMC Dev Biol. 9, 9, doi: 1471-213X-9-9 10.1186/1471-213X-9-9 (2009). 19200381 PMC2645379

[b51] Cox G. F. . Intracytoplasmic sperm injection may increase the risk of imprinting defects. Am J Hum Genet. 71, 162–164, doi: S0002-9297(07)60044-5 10.1086/341096 (2002). 12016591 PMC384973

[b52] Lawler S. D., Povey S., Fisher R. A. & Pickthall V. J. Genetic studies on hydatidiform moles. II. The origin of complete moles. Ann Hum Genet. 46, 209–222 (1982). 6214984 10.1111/j.1469-1809.1982.tb00713.x

[b53] Hubertus J. . Altered expression of imprinted genes in Wilms tumors. Oncol Rep. 25, 817–823, doi: 10.3892/or.2010.1113 (2011). 21174059

[b54] Bussey K. J. . SNRPN methylation patterns in germ cell tumors as a reflection of primordial germ cell development. Genes Chromosomes Cancer 32, 342–352, doi: 10.1002/gcc.1199 (2001). 11746975

[b55] Ariel I. . Relaxation of imprinting in trophoblastic disease. Gynecol Oncol. 53, 212–219, doi: S0090-8258(84)71118-8 10.1006/gyno.1994.1118 (1994). 8188082

[b56] Walsh C., Miller S. J., Flam F., Fisher R. A. & Ohlsson R. Paternally derived H19 is differentially expressed in malignant and nonmalignant trophoblast. Cancer Res. 55, 1111–1116 (1995). 7866996

[b57] Poaty H. . Genome-wide high-resolution aCGH analysis of gestational choriocarcinomas. PLoS One 7, e29426, doi: 10.1371/journal.pone.0029426PONE-D-11-06046 (2012). 22253721 PMC3253784

[b58] McMinn J. . Unbalanced placental expression of imprinted genes in human intrauterine growth restriction. Placenta 27, 540–549, doi: S0143-4004(05)00208-0 10.1016/j.placenta.2005.07.004 (2006). 16125225

[b59] Ingrosso D. . Folate treatment and unbalanced methylation and changes of allelic expression induced by hyperhomocysteinaemia in patients with uraemia. Lancet 361, 1693–1699, doi: S0140-6736(03)13372-7 10.1016/S0140-6736(03)13372-7 (2003). 12767735

[b60] Hoyo C. . Methylation variation at IGF2 differentially methylated regions and maternal folic acid use before and during pregnancy. Epigenetics 6, 928–936, doi: 16263 (2011). 21636975 10.4161/epi.6.7.16263 PMC3154433

[b61] Kato Y. . Role of the Dnmt3 family in de novo methylation of imprinted and repetitive sequences during male germ cell development in the mouse. Hum Mol Genet 16, 2272–2280, doi: 10.1093/hmg/ddm179 (2007). 17616512

[b62] Novakovic P., Stempak J. M., Sohn K. J. & Kim Y. I. Effects of folate deficiency on gene expression in the apoptosis and cancer pathways in colon cancer cells. Carcinogenesis 27, 916–924, doi: 10.1093/carcin/bgi312 (2006). 16361273

[b63] Junaid M. A. . Folic acid supplementation dysregulates gene expression in lymphoblastoid cells–implications in nutrition. Biochem Biophys Res Commun. 412, 688–692, doi: S0006-291X(11)01412-4 10.1016/j.bbrc.2011.08.027 (2011). 21867686

[b64] Steegers-Theunissen R. P. . Periconceptional maternal folic acid use of 400 microg per day is related to increased methylation of the IGF2 gene in the very young child. PLoS One 4, e7845, doi: 10.1371/journal.pone.0007845 (2009). 19924280 PMC2773848

[b65] Haggarty P. . Folate in pregnancy and imprinted gene and repeat element methylation in the offspring. Am J Clin Nutr 97, 94–99, doi: 10.3945/ajcn.112.042572ajcn.112.042572 (2013). 23151531

[b66] Chan K. C. . Hypermethylated RASSF1A in maternal plasma: A universal fetal DNA marker that improves the reliability of noninvasive prenatal diagnosis. Clin Chem. 52, 2211–2218, doi: 10.1373/clinchem.2006.074997 (2006). 17068167

[b67] Chim S. S. . Detection of the placental epigenetic signature of the maspin gene in maternal plasma. Proc Natl Acad Sci USA 102, 14753–14758, doi: 10.1073/pnas.0503335102 (2005). 16203989 PMC1253547

[b68] Poon L. L., Leung T. N., Lau T. K., Chow K. C. & Lo Y. M. Differential DNA methylation between fetus and mother as a strategy for detecting fetal DNA in maternal plasma. Clin Chem. 48, 35–41 (2002). 11751536

[b69] Chiu R. W. . Effects of blood-processing protocols on fetal and total DNA quantification in maternal plasma. Clin Chem. 47, 1607–1613 (2001). 11514393

[b70] Livak K. J. & Schmittgen T. D. Analysis of relative gene expression data using real-time quantitative PCR and the 2(-Delta Delta C(T)) *Method*. Methods 25, 402–408, doi: 10.1006/meth.2001.1262S1046-2023(01)91262-9 (2001). 11846609

[b71] Wojdacz T. K. & Dobrovic A. Methylation-sensitive high resolution melting (MS-HRM): a new approach for sensitive and high-throughput assessment of methylation. Nucleic Acids Res. 35, e41, doi: 10.1093/nar/gkm013 (2007). PMC1874596 17289753

[b72] Thakur S., Rahat B., Hamid A., Najar R. A. & Kaur J. Identification of regulatory mechanisms of intestinal folate transport in condition of folate deficiency. J Nutr Biochem. 26, 1084–1094, doi: 10.1016/j.jnutbio.2015.05.002S0955-2863(15)00135-7 (2015). 26168702

